# Supplementary figures and images for: Dendrite regeneration in C. elegans is controlled by the RAC GTPase CED-10 and the RhoGEF TIAM-1
Source: PLoS Genet. 2022 Mar 28;18(3):e1010127. doi: 10.1371/journal.pgen.1010127 (PMC8989329; doi:10.1371/journal.pgen.1010127)

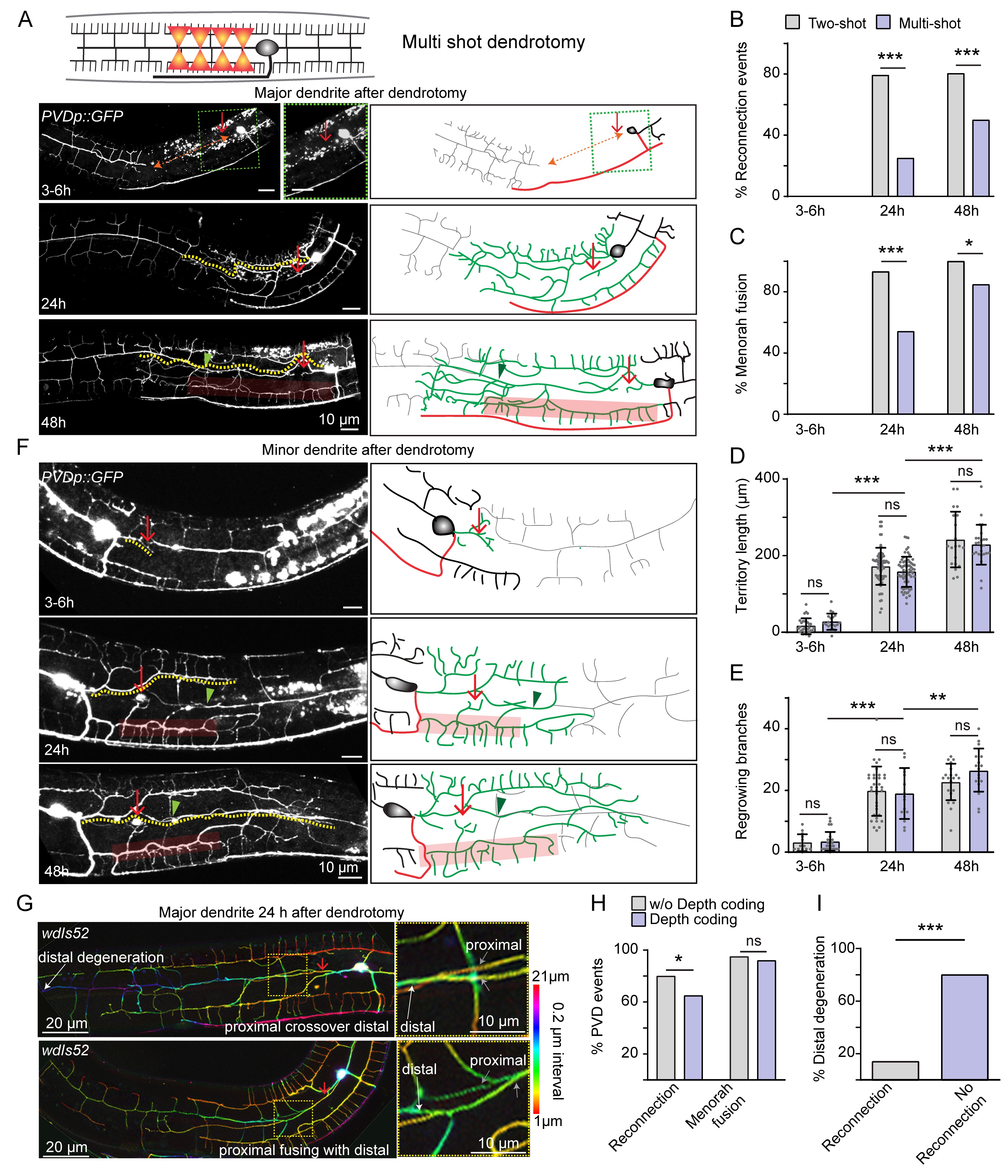

Supplement: S1 Fig — (A) The confocal images and illustrations (right) of regeneration phenomena of primary major dendrites of PVD at 3h, 24h and 48h post-dendrotomy using four laser shots. The experiment was performed in worms expressing wdIs52 (pF49H12.4::GFP) reporter. The large gap created at 3h post-dendrotomy due to multiple laser shots is indicated with orange dotted line with double-arrowheads (topmost panel). The faded red box and green arrowhead highlight the menorah-menorah fusion event and reconnection phenomenon, respectively. In the illustration, the regrowing dendrites, the remnants of distal part, and the axon is indicated in green, grey, and red colors, respectively. (B-C) The percentage occurrence of reconnection (B) and menorah-menorah fusion events (C) at 3h, 24h and 48 h after single or multi-shot laser-dendrotomy is represented. N = 3–5 independent replicates, n (number of regrowth events) = 20–60. (D-E) Quantification of territory length (D), N = 3–5 independent replicates, n (number of regrowth events) = 20–70 (D), and the total number of branches (E), N = 3–5 independent replicates, n (number of regrowth events) = 14–34 at 3h and 24h post-dendrotomy using single and multi-shots. (F) Confocal images with schematics showing the regeneration events at 3, 24, and 48h following the injury on primary minor dendrites. (G) Depth coded images of dendrite regeneration events in the wildtype worms along with magnified version of the reconnection area (right) showing the proximal part contacting the distal part or crossing over the distal part. (H) Quantification of reconnection and menorah-menorah fusion events counted from the depth-coded vs regular z-projected images. (I) Quantification of degeneration of the distal parts in the ’reconnection’ vs ‘no reconnection’ events. For, H-I, N = 3–4 independent replicates, n (number of regrowth events) = 20–30. Statistics, for B-C, Fisher’s exact test, taking p<0.05*, 0.001***. For D-E, one-way ANOVA with Tukey’s multiple comparis [file pgen.1010127.s001.tif]

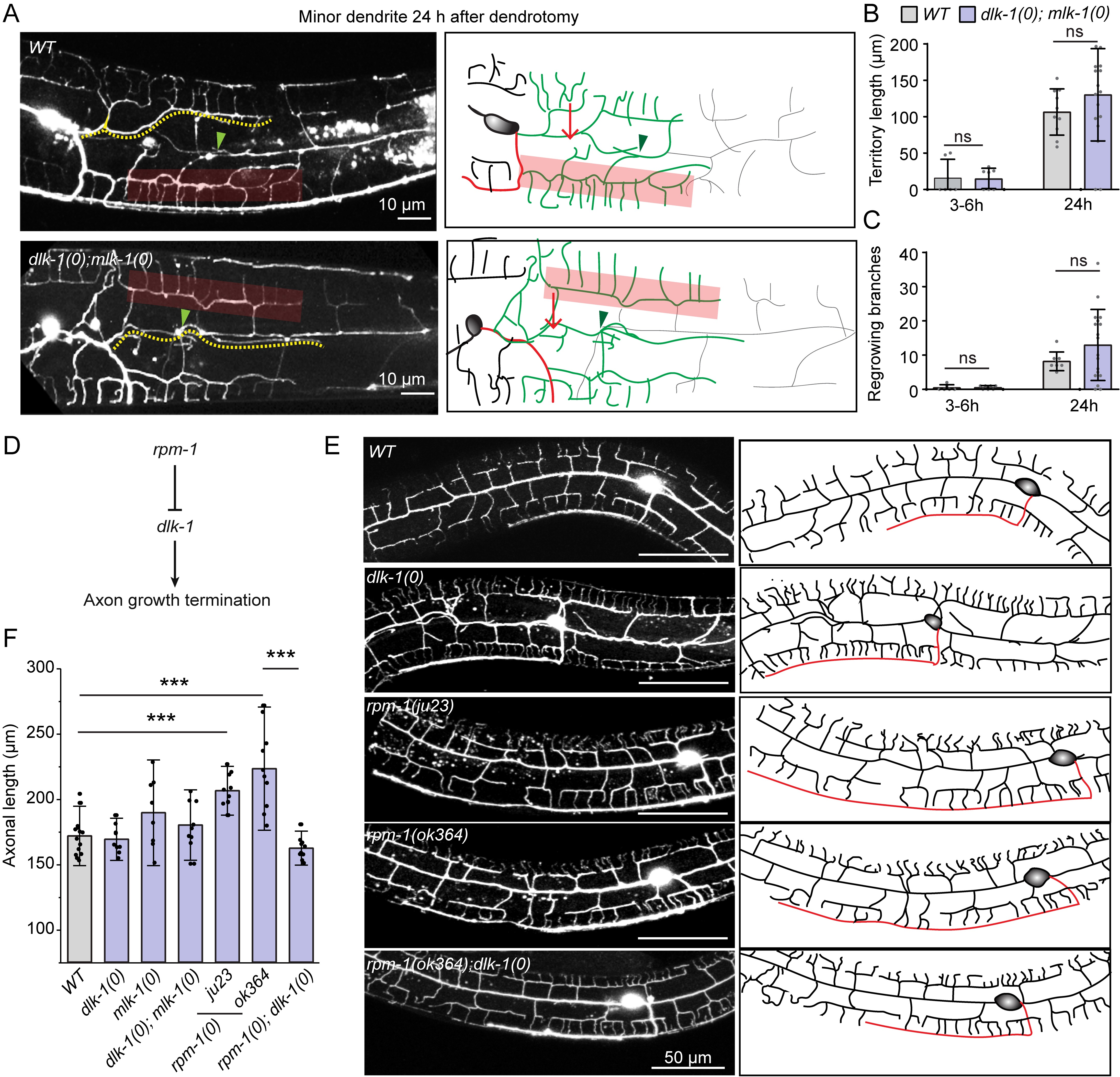

Supplement: S2 Fig — (A) Confocal images of the regeneration events of minor dendrites in wild-type and dlk-1(0);mlk-1(0) backgrounds at 24h post-dendrotomy. The schematics representing site of dendritic injury (red arrow), regenerated dendrite (green), distal part (grey), reconnection phenomena (green arrowheads) and menorah-menorah fusion (semi-transparent red boxes). Territory length is represented as yellow dotted lines in the confocal image. (B-C) The territory length (B) and the number of regrowing branches (C) at3-6h and 24h post-dendrotomy. N = 3 independent replicates, n (number of regrowth events) = 7–19. (D) The genetic pathway involving rpm-1, controlling axon growth termination. (E) Representative confocal images showing the developmental phenotype of PVD in various mutants in rpm-1 pathway. In the schematics, the axon in shown in red. Please note that in rpm-1 mutants, an overshooting of axon is noticed. (F) The quantification of axonal length of PVD neurons in the wild-type, dlk-1(0), mlk-1(0), dlk-1(0); mlk-1(0), rpm-1(ju23), rpm-1(ok364) and rpm-1(ok364);dlk-1(tm4024) mutants at L4 stage, N = 3–4 independent replicates, n (number of PVD imaged) = 8–27. Statistics, for B-C & F one-way ANOVA with Tukey’s multiple comparison method taking p<0.05*, 0.01**, 0.001***. Error bars represent SD. ns, not significant. (TIF) [file pgen.1010127.s002.tif]

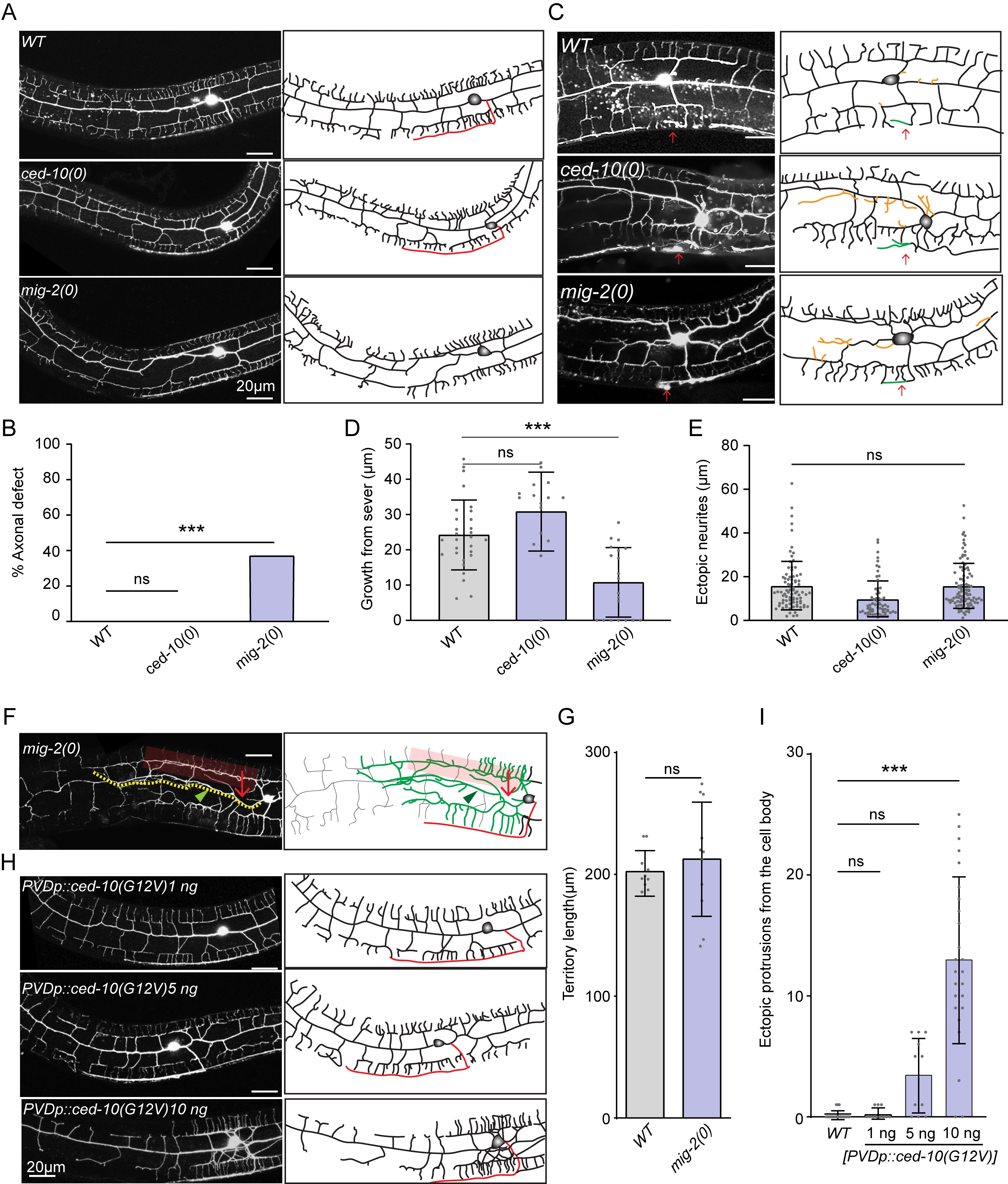

Supplement: S3 Fig — (A) Confocal images of PVD neuron in the wild-type, ced-10(0), and mig-2(0) is shown along with its illustrations (right) indicating the axon in red. (B) The axonal defect at L4 stage is calculated as percentage defect. N = 3 independent replicates, n (number of PVD imaged) = 10–12. (C) Confocal images of axon regeneration events in the wild-type, ced-10(0) and mig-2(0) at 24h post-axotomy along with their schematics indicating site of axonal injury with red arrow, regrowing axon from severed end in green and ectopic neurites in orange color. (D-E) Quantification of axon regeneration as growth from the severed end (D) and length of ectopic neurites (E) in the wild-type, ced-10(0) and mig-2(0) at 24h post-axotomy. N = 3–4 independent replicates, n (number of regrowth events) = 14–25. (F) Confocal image of dendrite regeneration in mig-2(0) at 24h post-dendrotomy. The illustration indicating the regrowing dendrites in green color, distal part in grey color, reconnection phenomenon with green arrowheads, menorah-menorah fusion with faint red rectangular boxes. (G) The territory length in the wild-type and mig-2(0) at 24h post-dendrotomy, N = 3–4 independent replicates, n (number of regrowth events) = 10–11. (H) The confocal images of PVD neuron at L4 stage expressing pser2prom3::ced-10(G12V) extrachromosomal transgenes. The pser2prom3::ced-10(G12V) plasmid was injected at 1ng/μl, 5ng/μl, and 10ng/μl concentrations to obtain these lines. (I) Quantification of number of ectopic neurites emerging out of cell body or adjacent dendrites at L4 stage in the wild-type and transgenic background expressing pser2prom3::ced-10(G12V) extrachromosomal arrays, N = 3 independent replicates, n (number of PVD imaged) = 11–20. Statistics, for B, Fisher’s exact test, for D-E & H, one-way ANOVA with Tukey’s multiple comparison method, and for G, unpaired t test, p<0.05*, 0.01**, 0.001***. Error bars represent SD. ns, not significant. (TIF) [file pgen.1010127.s003.tif]

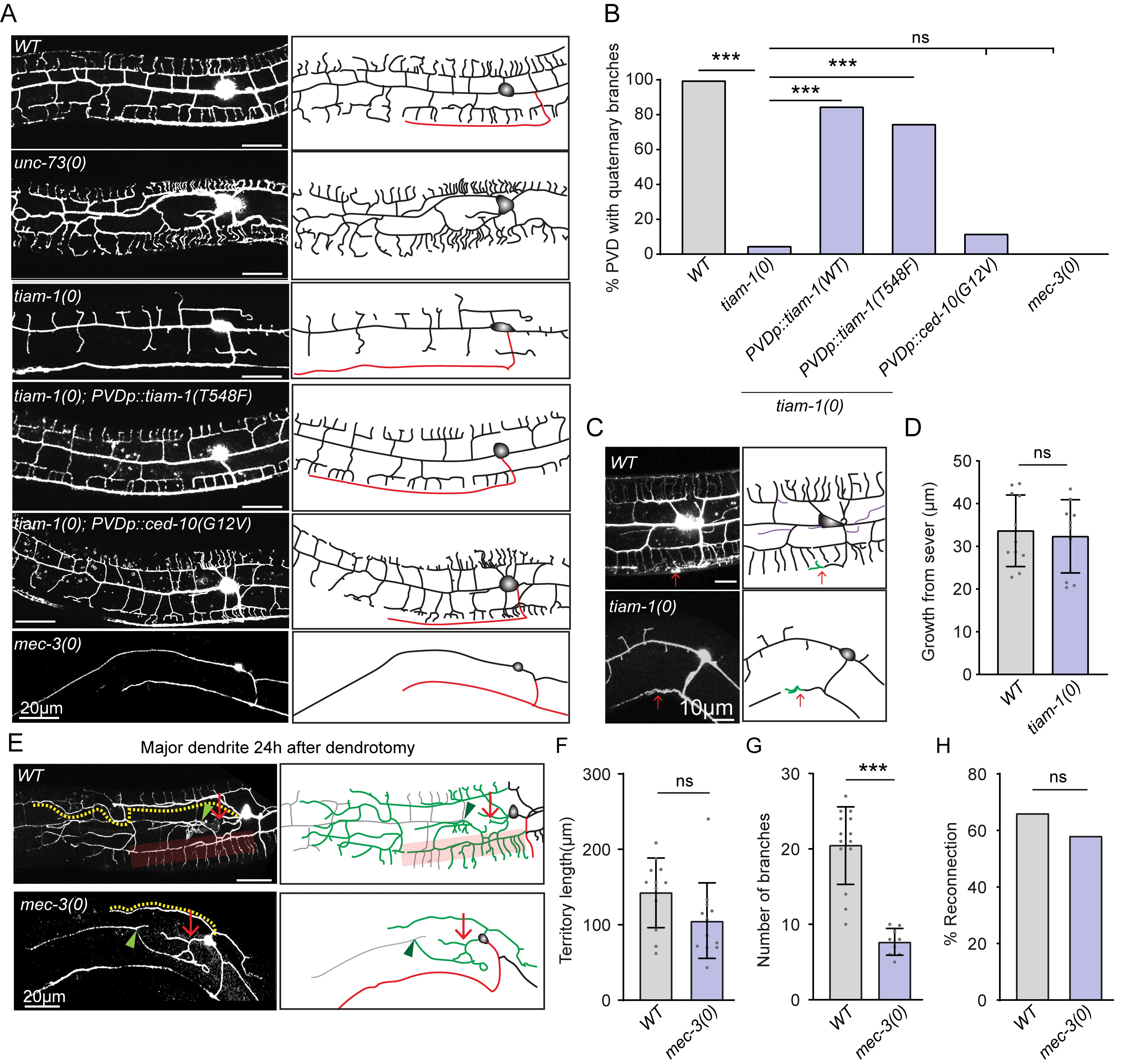

Supplement: S4 Fig — (A) Confocal images of PVD neuron in the wild-type, unc-73(0), tiam-1(0), pser2prom3::tiam-1(T548F);tiam-1(0), pser2prom3::ced-10(G12V);tiam-1(0), and mec-3(0) background at L4 stage. (B) The percentage of PVDs showing quaternary branches in the wild-type, tiam-1(0), pser2prom3::tiam-1(T548F);tiam-1(0), pser2prom3::ced-10(G12V);tiam-1(0) and mec-3(0) backgrounds, N = 3 independent replicates, n (number of PVD imaged) = 12–15. (C) Confocal images of axon regeneration at 24h post-axotomy in the wild-type and tiam-1(0) backgrounds. The regenerated axon from the severed end is shown in green color in the illustration. (D) The quantification of axon regrowth from the severed end in the wild-type and tiam-1(0), N = 3 independent replicates, n (number of regrowth events) = 10–12. (E) The confocal images of dendrite regeneration at 24h post-dendrotomy in the wild-type and mec-3(0) is shown along with their schematics representing the site of injury (red arrow), regenerated dendrites (green), reconnection events (green arrowhead), and the menorah-menorah fusion event (faint red rectangular box). (F-G) The territory length (F) and the number of regrowing branches (G) in the wild-type and mec-3(0) at 24h post-dendrotomy, N = 3 independent replicates, n (number of regrowth events) = 11–14. (H) Percentage of worms showing reconnection phenomena at 24h post-dendrotomy in the wild-type and mec-3(0), N = 3 independent replicates, n (number of regrowth events) = 11–14. Statistics, for, B & H, Fisher’s exact test, for D & F-G, unpaired t test considering p<0.05*, 0.01**, 0.001***. Error bars represent SD. ns, not significant. (TIF) [file pgen.1010127.s004.tif]
