# Supplementary material for: Dendrite regeneration in C. elegans is controlled by the RAC GTPase CED-10 and the RhoGEF TIAM-1
Source: PLoS Genet. 2022 Mar 28;18(3):e1010127. doi: 10.1371/journal.pgen.1010127 (PMC8989329; doi:10.1371/journal.pgen.1010127)
Supplement: Attachment — Submitted filename: Response Letter-Revision-310122.pdf [file pgen.1010127.s007.pdf]

Anindya Ghosh Roy, PhD
Associate Professor, Wellcome Trust-DBT Intermediate Fellow

National Brain Research Center (Deemed University)
(An Autonomous Institute of Deptt. of Biotechnology, Ministry of Science and Technology, Govt. of India)
NH-8, Nainwal Mode, Manesar-122051 (Haryana)

Point wise response

Dated, 30th Jan 2022, Gurgaon

Re: Dendrite regeneration in C. elegans is controlled by the RAC GTPase CED-10 and the RhoGEF TIAM-1 (PGENETICS-D-21-01021R1)

We thank the editors to consider our manuscript further for the review process. We thank the reviewers for their valuable inputs for the betterment of the manuscript. We have addressed all the points clearly and new experimental results were provided wherever suggested.

Reviewer #1: This paper uses powerful genetic and live cell imaging approaches to screen for effectors of dendritic regeneration. The experimental strategy exploits the stereotypical and readily visible dendritic branching pattern of the PVD nociceptive neuron in C. elegans. For these experiments, the PVD neuron is labeled with GFP and a laser is used to sever the primary PVD dendrite. Extensive dendritic regeneration was quantified from images collected a later time points. An extensive list of available mutants derived from studies of axon regeneration were tested for potential roles in dendrite regeneration. Although results are largely negative, these findings are important because they are consistent with the emerging evidence that axonal and dendritic regeneration are likely to rely on distinct mechanisms. A key finding of this work is the discovery that mutations that disable conserved effectors of actin dynamics, CED-10/RAC and TIAM-1/GEF, also impair PVD dendrite regeneration. Interestingly, rescue experiments point to both cell autonomous (PVD) and non-cell autonomous (epidermis) roles. A constitutively active version of ced-10 rescues the tiam-1(0) regeneration defect, a finding consistent with previous work showing that TIAM-1 functions as a GEF (guanine exchange factor) to activate CED-10. The experiments are rigorous, the paper is well-written (see minor revisions), and new findings are significant. Additional experiments are necessary, however, to shore up the proposed role of TIAM-1 in the regeneration mechanism as outlined below.

Response: We thank you for your appreciation of the work. We have addressed all the concerns raised here and provided additional data to strengthen the specific role of TIAM-1 GEF in dendrite regeneration.

Telephone: +91-124-2845 237 Fax : +91-124-2338910
Email : anindya@nbrc.ac.in **Website : www.nbrc.ac.in**

Anindya Ghosh Roy, PhD
Associate Professor, Wellcome Trust-DBT Intermediate Fellow

National Brain Research Center (Deemed University)
(An Autonomous Institute of Deptt. of Biotechnology, Ministry of Science and Technology, Govt. of India)
NH-8, Nainwal Mode, Manesar-122051 (Haryana)

Major Revisions

1. PVD dendritic branching is drastically reduced in tiam-1 mutants which show limited secondary and tertiary branch outgrowth. It thus seems plausible that the overall reduction in PVD dendritic branching in tiam-1 mutants could also hinder regeneration and that this effect would also be observed for mutants in other genes (e.g., hpo-30, lect-2, act-4, dma-1) that drive PVD branching. The authors need to test at least one additional PVD dendritic branching mutant to rule out this possibility.

Response: We agree with Reviewer #1 on the above point that the effect on dendrite regeneration in the *tiam-1* mutant could be influenced by the dendrite branching defect in this mutant. Thus we checked dendrite regeneration phenotype in a few mutants with developmental branching defect in PVD. For example, the *hpo-30(ok2047)* mutant showed a developmental defect in PVD dendrites (*Tang et al 2019, eLIFE*). In *hpo-30* mutant, the extent of regeneration (territory length) was reduced as similar to *tiam-1* mutant (13<n<15, N>2, unpaired student t test, considering $p<0.05^{*}$, 0.01^{**}, 0.001^{***}).

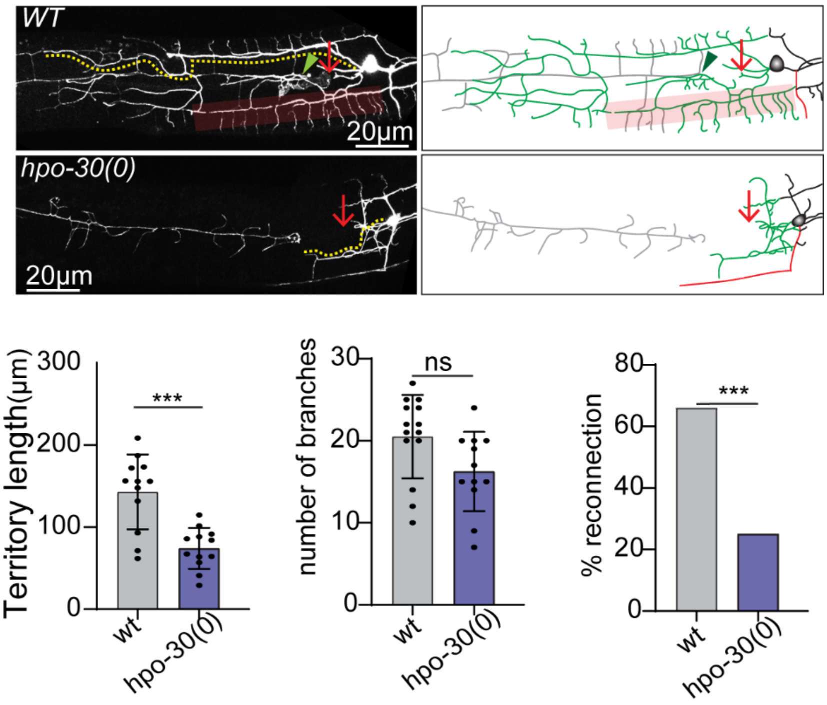

hpo-30 acts upstream to *tiam-1* in developmental branching by controlling actin dynamics (*Tang et al 2019, eLIFE*). It is possible that regeneration of PVD dendrites may utilize a similar mechanism for the new regrowth thus dependent on *hpo-30* upstream to *tiam-1*.

Additionally, we tested the *mec-3* mutant, in which only primary dendrite are present (Figure S4A-B,) (*Smith et al 2013 Neuron*). To our surprise, unlike the *hpo-30* dendrite arborization mutant, there was a robust regrowth response from the primary dendrite following dendrotomy. Both the territory length and reconnection frequency with the distal dendrite were similar to the wild type control (Figure S4 E-F). Though the number of regrowing branches was reduced, the overall regenerative capacity of the mec-3 mutant neuron was comparable to the wildtype (Fig S4F). Therefore, this addresses the point that the

Telephone: +91-124-2845 237 Fax : +91-124-2338910
Email : anindya@nbrc.ac.in **Website : www.nbrc.ac.in**

Anindya Ghosh Roy, PhD
Associate Professor, Wellcome Trust-DBT Intermediate Fellow

National Brain Research Center (Deemed University)
(An Autonomous Institute of Deptt. of Biotechnology, Ministry of Science and Technology, Govt. of India)
NH-8, Nainwal Mode, Manesar-122051 (Haryana)

mutants with diminished higher-order branches are not necessarily compromised in their potential for dendrite regeneration. Moreover, to find the specific role of *tiam-1* in dendrite regeneration, we expressed the version of TIAM-1 with compromised GEF activity (TIAM-1 T548F). This mutant version could rescue the developmental branching phenotype (Figure S4A-B) as seen before (Tang et al., eLIFE, 2019), but failed to rescue the dendrite regeneration phenotype in the *tiam-1* mutant. This indicated a specific role of TIAM-1 in dendrite regeneration. Please see the Line 344-357, Page 16 in the revised manuscript.

2. If TIAM-1 GEF activity is required for activating CED-10-dependent regeneration, then a tiam-1 point mutation that specifically eliminates TIAM-1 GEF activity should impair dendritic regeneration (Demarco et al., 2012). This question is important because a recent paper showed that TIAM-GEF activity is apparently not required for PVD dendritic branching (Tang et al., eLIFE, 2019).

Response: We generated the GEF dead *tiam-1* transgene (T548F) in *tiam-1(0* mutant background, which rescues the dendrite arborization defect in the *tiam-1(0)* mutant (Figure S4A-B) as seen before (Tang et al., eLIFE, 2019). However, the regeneration defects including the reduced territory length and the number of branches were not rescued by this transgene in *tiam-1(0)* mutant. This indicated that the GEF activity of TIAM-1 is required for dendrite regeneration (Figure 6A-B). This also strengthened the hypothesis that the impaired dendrite regeneration in the *tiam-1* mutant is not a secondary effect of the aberrant dendrite arborization in this mutant. Please see the Line 350-357, Page 16 in the revised manuscript.

3. The authors report that severed PVD dendrites regrow and ultimately fuse with each other to restore a contiguous dendritic arbor. The evidence of fusion is limited to the observation that the tips of apposing regenerated, GFP-labeled dendrites appear continuous in the light microscope. This observation does not rule out the alternative explanation that the regenerated dendrites are overlapping each other or touching but not actually fused. I'm not requiring an experiment to distinguish between these possibilities since this does not seem be a convention in the field but the authors need to address this caveat at the very least in the manuscript. This question is actually quite significant since the long term goal of this work on regeneration in model organisms is to discover pathways that can restore function to injured circuits.

Response: We concur with the reviewer that it is difficult to determine correctly whether the proximal and distal parts of the injured dendrites fuse during regeneration. For this reason, we described the contacts between the proximal and distal primary dendrites as 'reconnection' events. To address whether the two tips actually contact or appeared to be connected in the Z-projected image, we represented the confocal planes in the Z-projected image as depth-coded images. We made sure that the contact between the proximal and distal dendrites are at the same optical depth while counting the event as 'reconnection'. This way, in a false reconnection event, the proximal and distal end would

Telephone: +91-124-2845 237 Fax : +91-124-2338910
Email : anindya@nbrc.ac.in Website : www.nbrc.ac.in

Anindya Ghosh Roy, PhD
Associate Professor, Wellcome Trust-DBT Intermediate Fellow

National Brain Research Center (Deemed University)
(An Autonomous Institute of Deptt. of Biotechnology, Ministry of Science and Technology, Govt. of India)
NH-8, Nainwal Mode, Manesar-122051 (Haryana)

appear in different colors i.e. at different optical planes (Fig S1G). By this method, 60% of events were counted as 'reconnection events' as opposed to 80% counted when the projected image was not depth-coded (Fig S1H).

We have now revised the quantification of the reconnection events based on their respective depth-coded images. Another correlation that has been considered into account is that in the cases of false reconnection events, the distal part of the dendrites tends to degenerate (Figure S1I). Similarly, the menorah-menorah fusion events (Figure S1H) were also judged in this procedure. Please see the Line 128-138, Page 6-7 in the revised manuscript. Also please see the method section "Dendrite Regeneration Analysis and Quantification" line 512-522, page 24 in the revised manuscript.

Minor revisions

1. Minor grammatical and stylistic errors are scattered throughout the text. The use of the article "the" is problematic in several instances (e.g., "triggers elevation in the Cyclic Adenosine Monophosphate (cAMP)…" should be "triggers elevation of Cyclic…"

Response: We have incorporated the changes that you have suggested and reduced the usage of "the" wherever inappropriate. We have revised our manuscript text to avoid the occurrence of any grammatical errors to the best of our abilities.

2. Top of page 9: what does "multivariate process" mean?

Response: We used this terminology to address the fact that the dendrite regeneration process can be accessed using different variables such as 'territory length', 'Branch length', 'Branch number', 'Reconnection events', 'Menorah-Menorah fusion' and any of these variables can get affected in a given mutant. These different variables together define the dendrite regeneration in our study. We have now revised this sentence slightly to make it more explicit. The revised sentence is "Our results showed that the dendrite regeneration involves multiple cellular processes comprising regrowth, branching, and fusion events independent of conventional axon regeneration pathways, including DLK-1/MLK-1. Please see Line 105-108 , Page 5 in the revised manuscript.

3. Top of page 14: what does this mean? "Showed a significant decrease in the same."

Response: We wanted to address the point that the length of ectopic neurites emerging from nearby dendrites after axotomy is reduced in the double mutant of *dlk-1* and *mlk-1* but the single mutants did not

Telephone: +91-124-2845 237 Fax : +91-124-2338910
Email : anindya@nbrc.ac.in Website : www.nbrc.ac.in

Anindya Ghosh Roy, PhD
Associate Professor, Wellcome Trust-DBT Intermediate Fellow

National Brain Research Center (Deemed University)
(An Autonomous Institute of Deptt. of Biotechnology, Ministry of Science and Technology, Govt. of India)
NH-8, Nainwal Mode, Manesar-122051 (Haryana)

show any effect. This part is rewritten in the main text for a better understanding. Line 235-236, Page 11 in the revised manuscript.

4. Pg 18 suggested rewording: "dendrite regeneration assay indicated that most known effectors of axon regeneration are not required for dendrite regeneration in PVD neurons."

Response: Thanks for this suggestion and it has been incorporated into the main text. Line 278-280, Page 13 in the revised manuscript.

5. Pg 20 What is the meaning of this sentence? "The number of filopodia like structures (arrowheads, Figure 5A) and territory covered seemed to have decreased in ced-10(0) as compared to wild type…" Figure 5C shows a significant effect for "territory covered" but Figure 5D shows no significant effect for "regrowing branches."

Response: The observation regarding this image was that there are filopodia-like outgrowths indicated by the red arrowheads at 6h post-dendrotomy in Figure 5A. These were quantified as the number of branches in Fig 5D. In our revised manuscript, the number of branches is significantly reduced in the *ced-10* mutant at 6 h as compared to the wildtype neurons(Figure 5D). With an increase in the sample number, the data has become more statistically relevant. We have rewritten this portion in the main text for more clarity. Please see Line 295-298 , Page 14 in the revised manuscript.

6. Pg 20. What does "higher concentration lines" mean and why is this notable?

Response: Expression level of extrachromosomal array transgenes depends on the injected concentration of DNA. When the activated form of CED-10 G12V was injected at same concentration (10ng/uL) as the wild type form of CED-10, it led to an over-branching phenotype near the cell body (Figure S3H). Therefore, we have injected the CED-10-G12V (constitutively active) construct at various concentrations to get transgenes that caused a milder developmental phenotype. We used these low concentration lines (5ng/ul) for dendrotomy experiments to understand the role of constitutively active CED-10 in the process. We have mentioned the concentration of the injection while mentioning the low or high concentration lines. Please see Line 311-312, Page 14 in the revised manuscript.

7. Pg 21, "This infers…" should be "This suggests…"

Response: We thank Reviewer #1 for the suggestion. It is now corrected in the main text. Please see Line 321-323, Page 15 in the revised manuscript.

Telephone: +91-124-2845 237 Fax : +91-124-2338910
Email : anindya@nbrc.ac.in **Website : www.nbrc.ac.in**

Anindya Ghosh Roy, PhD
Associate Professor, Wellcome Trust-DBT Intermediate Fellow

National Brain Research Center (Deemed University)
(An Autonomous Institute of Deptt. of Biotechnology, Ministry of Science and Technology, Govt. of India)
NH-8, Nainwal Mode, Manesar-122051 (Haryana)

8. Figure 1 The yellow dotted line is very difficult to see. Needs to be brighter with thicker dots.

Response: We acknowledge this suggestion and have made the dotted line much bolder for better visibility. Please see the revised figures.

9. Figure 3. Images of the mCherry::RAB-3 marker (Panel B at 24h and 48h) are unconvincing given large number of fluorescent puncta that seem to be distributed throughout the field of view?

Response: We observe a significant amount of autofluorescence punctae from the gut that are visible in both the green and red fluorescence channels. We have revised this experiment with a strategy to remove the autofluorescence puncta in the background. We have provided the newly acquired images with background correction using auto-fluorescence acquired in the near UV channel. Please see the revised Figure 3B.

Reviewer #2: In this manuscript Brar et al. perform a basic characterization of dendrite regeneration in C. elegans PVD neurons, compare dendrite and axon regeneration in the same cell, and identify two genes required for dendrite regeneration. This work provides an important foundation for understanding mechanisms that allow neurons to respond to dendrite injury. Dendrite regeneration (with the exception of fusion, which is specific to C. elegans) has been studied almost exclusively in Drosophila. Having another model system in which to study this process is a substantial advance. As in Drosophila, none of the core axon regeneration machinery regulates dendrite regeneration, and these are compared in the same cell. Moreover, the authors find two regulators of dendrite regeneration. This is a nice foundational story that provides good footing to use C. elegans to investigate dendrite regeneration. There are just a few points that would strengthen the manuscript further:

Response: Thanks for appreciating the strength of this study. We have addressed all the points below.

1. Axon regeneration in PVD looks quite subtle; growth difficult to see in control image in figure 3D. Perhaps a different image might help? It looks like the axon does not regrow to reach its former length; is this the case? If so, it would be good to discuss what this might mean for function.

Telephone: +91-124-2845 237 Fax : +91-124-2338910
Email : anindya@nbrc.ac.in Website : www.nbrc.ac.in

Anindya Ghosh Roy, PhD
Associate Professor, Wellcome Trust-DBT Intermediate Fellow

National Brain Research Center (Deemed University)
(An Autonomous Institute of Deptt. of Biotechnology, Ministry of Science and Technology, Govt. of India)
NH-8, Nainwal Mode, Manesar-122051 (Haryana)

Response: The axon regeneration response in PVD neuron following the axotomy at the ventral nerve cord was not as pronounced as observed in other neurons of *C. elegans* (*Wu et al 2007 PNAS, Yanik et al 2004 Nature*). Also, we did not observe any fusion with the distal portion. However, we observed other phenotypes like redirection of axonal cargoes like RAB-3 to the adjacent dendrites and formation of ectopic branches similar to regenerating axons in *Drosophila* da neurons (*Stone et al 2010 MBoC*). Since the point of axotomy is closer to the synaptic region, the response may be less as compared to the unfasciculated axons of other model neurons (*Wu et al 2007 PNAS, Yanik et al 2004 Nature*). When we performed axotomy near the cell body, we did not see the regrowth from the severed tip. Instead, we mostly observed ectopic process coming from the cell body and conversion of dendrites into axon-like processes similar to other systems in cases of axotomy near the cell body. Therefore, to study the axonal regrowth from the severed tip, we chose this paradigm.

Concerning the behavioral or functional aspect of PVD neuron, we have not studied behavioral response after axotomy or dendrotomy in great detail yet. Therefore, it is beyond the scope of this paper. We have added this point in the discussion that studying the functional outcome of axon and dendrite regeneration would a good future direction of this study. Pleasec see Line 400-402, Page 18 in the revised manuscript.

2. In the manuscript loss of function mutations are designated (0). Are these null alleles? For ced-10 it would be particularly helpful to know if the alleles used are null as no developmental phenotype is observed.

Response: We are using the symbol (0) to represent the loss of function mutations. These can either be deletion or substitution mutations which affect the functionality of the gene leading to a loss of function phenotype. The *ced-10*(*n3246*) allele has a Glycine 60 to Arginine mutation in the DTAG motif present in the binding site of the γ-phosphoryl group of GTP. This glycine residue is responsible for the GTP-induced conformational changes and is conserved among all GTPases. This mutation leads to a reduced signaling activity of the GTPase as per the information on the structure and function of GTPases (*Bourne et al., 1991 Nature*). This mutant allele of *ced-10* has been used as a loss of function allele in various studies before (*Reddien & Horvitz et al., 2000, Nature cell biology, Norgaard et al. 2018, Plos Genetics*). The null allele *tm597* (612bp deletion in *ced-10* gene) of *ced-10* leads to embryonic lethality (*Norgaard et al. 2018, Plos Genetics; Lundquist et al. 2001, Development*) and thus could not be used for dendrite regeneration studies.

3. It would be very helpful to show whether axon regeneration is affected similarly to dendrite regeneration in ced-10 and tiam-1 mutants. As the story stands, it is difficult to know whether either is required broadly for regenerative growth in this cell type, or specifically for dendrite regeneration.

Telephone: +91-124-2845 237 Fax : +91-124-2338910
Email : anindya@nbrc.ac.in **Website : www.nbrc.ac.in**

Anindya Ghosh Roy, PhD
Associate Professor, Wellcome Trust-DBT Intermediate Fellow

National Brain Research Center (Deemed University)
(An Autonomous Institute of Deptt. of Biotechnology, Ministry of Science and Technology, Govt. of India)
NH-8, Nainwal Mode, Manesar-122051 (Haryana)

Response: We had already presented the data associated with axon regeneration in the *ced-10(n3246)* mutant in Figure S3 C-E of the previous version of the manuscript. In the revised version, this data is shown in Figure S3(C-E). The axon regeneration is not affected in this mutant. This indicated that dendrite regeneration is specifically dependent on *ced-10*. Please see Line 311-312, Page 14 in the revised manuscript.

We have also added the new data involving axotomy in *tiam-1* mutant (Figure S4 C-D) which was also unaffected similar to the *ced-10* mutant. This indicated that *ced-10* and *tiam-1* dependent dendrite regeneration is not a general mechanism of neurite regeneration. Please see Line 340-342, Page16 in the revised manuscript.

Sincerely,

Anindya Ghosh Roy.

Telephone: +91-124-2845 237 Fax : +91-124-2338910
Email : anindya@nbrc.ac.in **Website : www.nbrc.ac.in**
